# Mouse Models for Deciphering the Impact of Homologous Recombination on Tumorigenesis

**DOI:** 10.3390/cancers13092083

**Published:** 2021-04-25

**Authors:** Gabriel Matos-Rodrigues, Emmanuelle Martini, Bernard S. Lopez

**Affiliations:** 1 Université de Paris, INSERM U1016, UMR 8104 CNRS, Institut Cochin, Equipe, Labellisée Ligue Contre le Cancer, 75014 Paris, France; gabriel.rodrigues@nih.gov; 2 Université de Paris and Université Paris-Saclay, Laboratory of Development of the Gonads, IRCM/IBFJ CEA, Genetic Stability, Stem Cells and Radiation, F-92265 Fontenay aux Roses, France

**Keywords:** homologous recombination, DNA repair, genomic instability, mouse model, tumorigenesis

## Abstract

**Simple Summary:**

Homologous recombination (HR) is a DNA repair pathway essential to genome stability and mutations in many HR genes are correlated with cancer predisposition. Transgenic mouse models are critical to establish HR factors as tumor suppressor genes. However, investigating the effects of suppressing HR genes in vivo is challenging because invalidation of most of them leads to embryonic lethality in mammals. To tackle this issue, elaborated alternative strategies have been developed. Here we review these alternative HR-defective mouse models and reveal the impact of HR defects on tumorigenesis. We highlight that the central HR factor, RAD51, has yet to be well characterized in vivo and, unlike most HR factors, its inactivation has not been associated with cancer predisposition, revealing what we call the “RAD51 paradox”. Finally, we discuss the use of mouse models to develop targeted cancer therapies as well as to understand the mechanisms of HR inactivation-driven tumorigenesis in vivo.

**Abstract:**

Homologous recombination (HR) is a fundamental evolutionarily conserved process that plays prime role(s) in genome stability maintenance through DNA repair and through the protection and resumption of arrested replication forks. Many HR genes are deregulated in cancer cells. Notably, the breast cancer genes *BRCA1* and *BRCA2*, two important HR players, are the most frequently mutated genes in familial breast and ovarian cancer. Transgenic mice constitute powerful tools to unravel the intricate mechanisms controlling tumorigenesis in vivo. However, the genes central to HR are essential in mammals, and their knockout leads to early embryonic lethality in mice. Elaborated strategies have been developed to overcome this difficulty, enabling one to analyze the consequences of HR disruption in vivo. In this review, we first briefly present the molecular mechanisms of HR in mammalian cells to introduce each factor in the HR process. Then, we present the different mouse models of HR invalidation and the consequences of HR inactivation on tumorigenesis. Finally, we discuss the use of mouse models for the development of targeted cancer therapies as well as perspectives on the future potential for understanding the mechanisms of HR inactivation-driven tumorigenesis in vivo.

## 1. Introduction

Homologous recombination (HR) is a molecular process highly conserved through evolution that plays prominent roles in genome plasticity. Indeed, the DNA repair function(s) and the outcomes of HR are essential not only to maintain genome stability but also to promote genome variability [1,2,3,4,5]. HR allows the repair of different types of DNA lesions, mainly DNA double-strand breaks (DSBs) and interstrand crosslinks (ICLs) [6,7]. Moreover, an important role of HR in genome stability maintenance is the protection and resumption of arrested replication forks. However, prolonged replication fork arrest leads to DSBs that can thus be processed by HR [8,9].

DSBs are generally considered the most toxic lesion. DSBs can be generated by endogenous stresses resulting from cellular metabolism, such as replication stress and reactive oxygen species (ROS), as well as from exogenous factors, such as ionizing radiation and chemotherapy agents (e.g., topoisomerase inhibitors). DSBs can also be programmed to trigger beneficial genomic rearrangements during meiotic differentiation [10] or the establishment of the immune system [11]. HR is also a driving force for the evolution of multigene families [12]. Therefore, thanks to this versatility, HR is involved in many fundamental biological processes. Finally, the technical application of HR constitutes the basis of targeted gene replacement for gene therapy as well as for the precise design of engineered organisms [13,14].

Genetic instability is a hallmark of aging and cancer [15,16,17]. Remarkably, markers of the DNA damage response have been found to be activated at pre-/early steps of tumorigenesis [18,19]. Since replication stress is a prominent endogenous source of DNA damage and genome instability, these data indicate a causal role of DNA replication stress in the early steps of tumor initiation [18,19,20]. Note that genetic instability can also fuel tumor progression. Because of its essential roles in genome stability maintenance, particularly in response to replication stress, HR is generally considered a tumor suppressor mechanism. Indeed, germline and somatic inactivating mutations in major HR actors have been observed in different types of tumors [21]. For example, germline heterozygous mutations in genes directly implicated in HR—*BRCA1, BRCA2*, *PALB2, RAD51C* and *RAD51D*—increase the risk of ovarian and breast cancer [22,23,24,25]. In addition, Fanconi anemia patients with biallelic mutations in *BRCA1, BRCA2* and *PALB2* show an increased risk of hematological malignancies and solid tumors [26,27,28,29,30,31,32,33].

Mouse models have been powerful tools to address cancer etiology and development in vivo. However, investigations on the effects of HR suppression in vivo have been challenging due to the fact that most HR genes are essential in mammals, and as such, their knockout leads to embryonic lethality. To tackle this issue, elaborated alternative strategies have been developed, and many mouse models have been designed to experimentally address the impact of HR defects on cancer development in vivo.

Here, we first summarize the molecular mechanisms of HR, presenting the roles of the main actors for which mouse models have been designed. Then, we present mouse models of HR deficiency and discuss how they contribute to the understanding of HR-driven tumorigenesis and the development of targeted therapies for HR-inactivated tumors.

## 2. Molecular Mechanisms of HR

**Figure 1:**
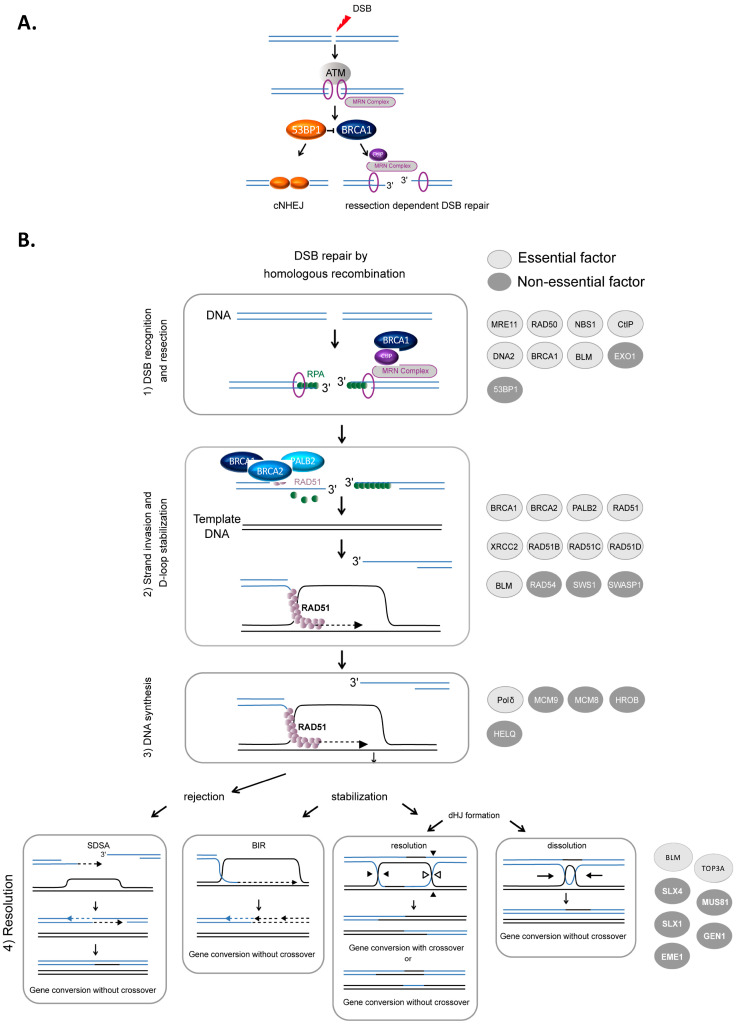
HR-mediated DSB repair. (**A**). Signaling and competition between cNHEJ and resection [34]. The involvement of 53BP1, BRCA1 and the MRN complex. (**B**). Model of DSB repair by HR. (1) The MRN complex initiates a 5′ to 3′ resection under the control of BRCA1/CtIP, generating a 3′ single-stranded DNA (ssDNA) (**A**). (2) RAD51 is then loaded to the 3′ single-stranded DNA by the combined action of BRCA2/PALB2 and BRCA1, resulting in the formation of an ordered RAD51-ssDNA nucleofilament. The invasion of homologous duplex DNA by the RAD51-ssDNA filament induces the formation of a displacement loop (D-loop). (3) The invading strand primes DNA synthesis. (4) The D-loop can be either dismantled, leading to DSB repair by synthesis-dependent strand annealing (SDSA) or stabilized, leading to either DSB repair by BIR or the formation of a double Holliday junction (dHJ). The dHJ can be resolved or dissolved, leading to a repair product either associated with a crossover event or not. The proteins involved in each corresponding step are listed on the right side of the figure. Light gray circles indicate essential factors; essential factors are defined as genes whose knockout leads to embryonic lethality in mice. Dark gray circles indicate nonessential factors; these are genes whose KO does not lead to embryonic lethality in mice.

HR-mediated DSB repair represents a paradigm for the molecular steps of HR. DSBs are first recognized and signaled by the MRN complex (MRE11/RAD50/NBS1) in cooperation with the ATM (ataxia-telangiectasia mutated) kinase and the chromatin remodeling machinery. Subsequently, DSBs can be either repaired by canonical nonhomologous end-joining (cNHEJ) or resected (Figure 1A) to then be repaired by HR or other resection-dependent DSB repair processes, such as single strand annealing (SSA) and alternative end-joining [34,35,36]. This choice between NHEJ and resection is controlled by the antagonistic activities of BRCA1, which favors resection, in competition with the protein complex 53BP1-Shieldin (53BP1-REV7-SHLD1-SHLD2-SHLD3), which prevents DNA end resection [37,38,39] (Figure 1A). The steps of HR that succeed DSB recognition can be summarized as follows (Figure 1B): (1) DSB resection to generate a 3′ ssDNA stretch (see Figure 1A); (2) RAD51 loading on the ssDNA, forming the ssDNA-RAD51 filament that promotes a search for homology and invasion of a homologous duplex DNA; (3) DNA synthesis primed by the invading 3′ end; and (4) the formation and resolution of HR intermediates that give rise to gene conversions (nonreciprocal exchange of genetic information) either associated with crossover or not (reciprocal exchange of adjacent sequences) (Figure 1B) (reviewed in [1,3,5]).

### 2.1. Resection

Resection, which is required to initiate HR, is performed in two steps (Figure 1A). BRCA1 is recruited to DSB sites via its interaction with NBS1 and associates with BARD1, forming an active E3 ubiquitin ligase. This complex ubiquitinates the endonuclease CtIP, which cooperates with MRE11 to initiate DSB resection [40,41]. Then, exonuclease 1 (EXO1) and/or the BLM/DNA2 (helicase/nuclease) complex extend the 3′ overhang [42,43,44,45] (Figure 1B). Finally, the 3′ ssDNA stretch created by resection is coated with replication protein A (RPA), protecting it (Figure 1B).

### 2.2. Loading RAD51 on ssDNA, Search for Homology and Strand Invasion

The loading of RAD51 onto ssDNA is performed by the BRCA2-PALB2 complex [46,47]. This protein complex interacts with BRCA1 and catalyzes the replacement of RPA by RAD51 on the stretch of 3′ ssDNA, creating the RAD51-ssDNA presynaptic complex [48,49,50]. Note that BRCA1 plays roles during different steps of HR: the initiation of resection and the loading of RAD51 (Figure 1B) [3,51,52].

The ssDNA-RAD51 filament scans the genome to search for homology. Once a homologous sequence is found, the filament invades the duplex homologous DNA and initiates strand exchange, creating a displacement loop (D-loop).

### 2.3. DNA Synthesis

The 3′ invading strand primes DNA synthesis through the recruitment of DNA and the copy of the invaded DNA molecule. Numerous studies have demonstrated the involvement of many polymerases in this process, although Polδ has been proposed to play a primary role [53]. The protein complexes HROB-MCM8–MCM9 and HELQ are proposed to have redundant helicase functions to promote DNA synthesis during HR [54].

### 2.4. Formation and Resolution of HR Intermediates

Strand invasion and DNA synthesis lead to the formation of different intermediates whose processing leads to gene conversion either associated with crossover products or not (Figure 1). The invading strand can be disassembled, channeling DSB repair toward synthesis-dependent strand annealing (SDSA) (Figure 1B). If stabilized, the D-loop can lead to DSB repair by break-induced repair (BIR) or to the formation of double Holliday junctions that can be either dissolved by the BLM-TOP3A-RMI1/2 complex or resolved by the structure-specific resolvases MUS81-EME1, GEN1 or SLX1 (Figure 1B) (reviewed in [2,55]).

Of note, the DNA helicase BLM, which is mutated in Bloom syndrome, plays several roles, sometimes contradictory, and at different HR steps. Indeed, BLM is involved in different steps of HR, including end resection at HR initiation [42,56], D-loop rejection and dHJ resolution at HR termination [56,57]. At resection initiation, depending on the cell cycle phase that modifies its interacting partners, BLM either favors the loading of 53BP1 on the DSB in G1 phase, preventing the initiation of unscheduled resection, or, in contrast, favors resection in S phase when interacting with TOP3 [58].

### 2.5. Accessory Proteins

RAD54, a member of the SWI2/SNF2 protein family (ATP-dependent chromatin remodelers), interacts with RAD51, and in vitro studies have proposed that it functions as a RAD51 cofactor [59,60,61]. RAD54 catalyzes the extension of joint molecules [62] and stabilizes the D-loop [63].

A family of six proteins (RAD51B, RAD51C, RAD51D, XRCC2, XRCC3, and RAD51AP1), known as the RAD51 paralogs (i.e., proteins that share sequence homology with RAD51 in a given species), has been identified in mammals. Two distinct complexes have been identified: RAD51B–RAD51C–RAD51D–XRCC2 (BCDX2) and RAD51C–XRCC3 (CX3) [64]. RAD51 paralogs favor the recruitment of RAD51 to DNA damage sites [65] and promote the formation and stabilization of the RAD51 nucleoprotein filament. However, the exact role of each paralog remains to be fully determined. In addition, RAD51 paralogs influence gene conversion tract length [66,67]. The SWSAP1 protein, a noncanonical paralog of RAD51, forms the so-called SHU complex when associated with SWS1 (SWSAP1-SWS1). SHU interacts with RAD51 and regulates its function [68].

## 3. Mouse Models of HR Deficiency

Molecular and cellular studies have detailed the molecular mechanisms of HR and its impact on genome stability maintenance in cultured cells, while clinical human genetic analyses have shown connections between HR defects and cancer development. The disruption of endogenous HR genes in mice should provide an extensive amount of information for understanding not only the molecular mechanisms of HR in vivo but also the etiology of cancer. Such models might constitute useful tools for the development of novel therapies for HR-deficient tumors.

### 3.1. HR Genes Are Essential in Mammals

Germline ablation of the pivotal HR player *Rad51* (see Figure 1B) leads to early embryonic lethality [69,70]. Soon after the discovery of the BRCA genes, many mouse models of germline genetic inactivation of *Brca1* and *Brca2* were generated using different gene-targeting strategies (Table 1). In all cases, homozygous deletion leads to embryonic lethality [71,72,73,74,75,76,77,78]. Note that homozygous inactivation of *BRCA1* and *BRCA2* is also proposed to be embryonic lethal in humans [79]. More generally, germline ablation of most other HR genes was also embryonic lethal in mouse models (Table 1).

This evidence from mice and humans suggests that in contrast to other models, such as yeast, zebrafish (*Danio rerio*) and flies (*Drosophila melano*), HR is essential in mammals [109,110,111,112]. However, there are a few mammalian HR genes that are not embryonic lethal (Table 2). Importantly, none of these genes control the central steps of HR: loading of RAD51, searching for homology and strand exchange. Based on these phenotypes, we define “essential” HR factors that lead to embryonic lethality when invalidated in mice (Figure 1).

### 3.2. Partial Loss of HR

To circumvent the embryonic lethality caused by the KO of “essential” HR factors, transgenic mice with partial loss of function of HR genes have been developed. A homozygous frameshift mutation in *BRCA1* called BRCA1-_AA2800_ that creates a truncated protein of 900 amino acids was reported in a patient with breast cancer [126]. Ludwig and colleagues generated a humanized mouse model of BRCA1-_AA2800_ called *Brca1^tr^*. *Brca1^tr/tr^* mice showed normal embryonic development and developed a variety of tumors in adulthood, with a high frequency of lymphomas and late onset breast carcinomas/adenomas [127]. This study shows that germline *Brca1* mutations can indeed lead to breast cancers without additional concomitant genetic manipulations (e.g., *Trp53* inactivation) in mice. However, *Brca1^tr/tr^* mice develop a variety of cancers different from those found in BRCA1-_AA2800_ human carriers.

Similar analyses have been carried out in mouse *Brca2* models. In humans, truncating mutations of exon 11 of *BRCA2* are associated with ovarian and breast cancer predisposition [128,129,130] (Breast Cancer Information Core, http://research.nhgri.nih.gov/bic/ accessed on 18 April 2021). The exon 11 of BRCA2 codes BRCT domains that are important for its interaction with RAD51 and, therefore, for HR [131,132,133]. Mice carrying truncating mutations of the exon 11 of *Brca2* show a high incidence of thymic lymphomas [134,135]. Although the type of tumors observed in humans and mice after disruption of the exon 11 of BRCA2 differ, in both species truncating mutations of the exon 11 of *Brca2* is a pro-tumorigenic event.

Many other mouse models of germline partial loss of function of *Brca1* and other HR genes have been generated (Table 3), and these studies established the importance of HR for tumor suppression in vivo.

### 3.3. Genetic Interactions Between HR Factors and other DNA Damage Response and Repair Genes

For most of the “essential” HR genes, including *Brca1*, *Brca2*, *Rad51*, *Palb2*, *Rad51c* and *Rad51l1/Rad51b*, germline co-inactivation of *Trp53* delays mouse death for a few days but remains embryonic lethal [70,73,99,103,105].

The knockout of the RAD51 paralog *Xrcc2* is an exception in which embryonic lethality occurs at later stages compared to the other “essential” HR genes (Table 1). The double knockout of *Xrcc2* and *Trp53* rescues embryonic lethality but leads to the development of multiple tumor types, including lymphomas, skin tumors, sarcomas and medulloblastomas, during early adulthood (~10 weeks), illustrating the fact that disrupting HR may induce a broad range of tumors [102].

However, several lines of evidence suggest that specific types of tumors seem to be related to which specific HR gene is affected and how. Using specific transgenic mouse models allowed a different landscape of tumor predisposition to be highlighted, depending on the step at which HR is disrupted. For example, mutations affecting the loading of RAD51 show a strong predisposition to lymphomas, whereas mutation of *Brca1*, which affects resection, induces a larger type of tumor [88]. Mouse models of *Brca2* partial loss of function also show increased tumorigenesis, with a particular predisposition to lymphomas [134,146]. Recently, two independent studies have developed *Brca1* mouse models that disrupt the interaction between BRCA1 and PALB2 [141,142]. These mutations abrogate the RAD51-loading function of BRCA1 while maintaining intact BRCA1-dependent control of DSB resection. These mice were born at low frequencies and showed a particular predisposition to the development of lymphomas [141,142]. Therefore, inactivation of *Xrcc2*, *Brca2* or the RAD51-loading function of BRCA1 leads to the formation of different types of tumors compared to *Brca1* mouse models that, in addition, abrogate DSB resection, showing that different modes of tumorigenesis exist depending on how HR is invalidated.

The genetic interaction between *Brca1* and *53BP1* (*Trp53bp1* in mouse) has been uncovered using the *Brca1^Δ11^* mouse model, which lacks exon 11 and therefore does not include a region necessary for the DNA end resection activity of BRCA1 [141,147]. *Brca1^Δ11^* homozygous mice are embryonic lethal, a phenotype that is completely abolished by concomitant *Trp53bp1* deletion [147]. The double-inactivated mice present normal HR efficiency and normal development and do not show increased tumorigenesis, highlighting that the rescue of DNA end resection in the *Brca1^Δ11^* mutant by deletion of *Trp53bp1* is enough to reverse most phenotypes [147]. In contrast, the *Tr53bp1* and *Brca1* double knockout mice that are proficient in DNA end resection but defective for RAD51 loading have reduced lifespan, and the mice that survive to adulthood develop lymphomas. As expected, a similar phenotype is found in *Brca1* mutants, such as *Brca1 ^L1363P/L1363P^* and *Brca1 ^CC/CC^*, which carry mutations affecting the BRCA1-PALB2 interaction and therefore are deficient in RAD51 loading [141,142,148]. Interestingly, *Brca1^CC/∆11^* mice, which carry the combination of two allelic mutations affecting RAD51 loading and DNA end resection, did not develop tumors and had a normal lifespan [141]. Together, these data suggest that the BRCA1-delta11q isoform of BRCA1, which maintains its RAD51 loading activity, provides further evidence that inactivation of BRCA1-mediated RAD51 loading fuels lymphoma development in mice.

The genetic interactions between *Brca1* and *Tr53bp1* highlight once again that different modes of tumorigenesis are observed depending on the step at which HR is disrupted (Table 4).

### 3.4. Tissue-Specific Ablation of HR and Targeted Cancer Therapy

Tissue-specific inactivation of HR genes is a powerful tool for in vivo cancer studies. Tissue-specific *Brca1* knockout in breast progenitors leads to breast tumor formation [154]. In addition, inactivation of the tumor suppressor *Trp53* stimulated tumorigenesis resulting from *Brca1* invalidation in mice [155]. Importantly, *TP53* mutations are frequently found in BRCA1 tumors [156,157]. Remarkably, *Brca1/Trp53* double-inactivated tumors share common histopathological characteristics with human BRCA1 breast cancers, as they are also highly proliferative, estrogen receptor (ER)-negative carcinomas that push borders, express basal epithelial markers, and exhibit a high degree of genomic instability [155]. For these reasons, mouse models with tissue-specific knockout of *Brca1* and *Trp53* have been extensively used in preclinical investigations [158,159,160,161,162]. Other mouse models have also revealed tumor predisposition upon tissue-specific inactivation of the RAD51 loading factors *Palb2* and *Brca2* [97,100].

Fanconi anemia patients carrying *BRCA2* mutations (FA-D1) also show predisposition to medulloblastoma brain tumors [163,164]. In agreement with a conserved tumor suppressor role of BRCA2 in the brain, *Brca2* and *Trp53* conditional inactivation in mouse neural progenitors leads to brain tumor formation, including medulloblastoma [165,166]. Curiously, unlike what was seen with BRCA2 loss, *Trp53* and *Brca1* codepletion in neural progenitors fully rescued the brain phenotypes of *Brca1* inactivation alone, and the authors did not report brain tumors [167]. This evidence from tissue-specific knockout of HR factors reinforces the idea that different modes of tumor initiation depend on which step of HR is affected and suggests a special BRCA2 function in the inhibition of brain tumor development. In addition, studies using medulloblastomas derived from the abovementioned *Brca2* mouse model pointed to complex genomic rearrangements called chromothripsis as a source of the genetic modifications leading to tumorigenesis in these mice [168].

Poly(ADP-ribose) polymerase (PARP) proteins play important roles during single-strand break repair and participate in the recruitment of the DNA repair machinery after break formation [169,170]. HR-deficient cells are hypersensitive to PARP inhibition, and this characteristic has been explored for potential development of BRCA-/HR-deficient cancer treatments [171,172,173]. Soon after the publication of the synthetic lethality of PARP inhibitors and HR deficiency, Rottenberg and colleagues explored the use of PARP inhibitors for cancer treatment in vivo using a *Brca1* mouse model of breast tumors [174]. The authors observed that PARP inhibition decreased tumor growth and increased mouse survival in vivo [174]. Since then, PARP inhibitors have been extensively used in clinical trials, and in 2014, they were approved for maintenance monotherapy in BRCA ovarian tumors that responded to platinum-based treatment [21]. However, acquired resistance is a major issue associated with the use of PARP inhibitors in the clinic [173,174]. PARP inhibitors create selective pressure on new mutations that can restore HR and, thereby, new clones are able to proliferate in the presence of PARP inhibitors and reconstitute tumors [173,175]. Interestingly, Rottenberg and colleagues previously observed such acquired resistance to PARP inhibitors in their mouse models in 2008 [174].

Secondary BRCA mutations restoring HR activity are a common pathway driving resistance to PARP inhibition [173,175]. Recent studies have described additional modes of restoring replication fork stability and/or HR in chemoresistant BRCA cells [173,175,176]. Not surprisingly, BRCA1 and BRCA2 tumors do not develop the same mechanisms of resistance to PARP inhibition [173,175]. Mutations in the 53BP1-sheldin-RIF1-REV7 pathway members generate PARP inhibitor resistance in BRCA1 cells but not BRCA2 cells [177,178,179,180]. Using a *Brca2* mouse model of breast tumors, Gogola and colleagues showed that inactivation of poly(ADP-ribose) glycohydrolase (PARG), which catalyzes PARP poly(ADP–ribose) degradation, is another mechanism of acquired resistance to PARP inhibition [181,182]. Therefore, mouse models of HR deficiency are not only important tools for the development of new targeted cancer treatment strategies but can also help elucidate the mechanisms of resistance to therapy.

## 4. Concluding Remarks and Perspectives

Transgenic mouse models have been key allies in the development and understanding of targeted therapy for HR-deficient tumors. They revealed that HR genes play essential roles in the suppression of tumorigenesis and showed that the modality of tumor predisposition depends on how HR is disrupted. However, to establish a direct relationship between HR disruption and cancer predisposition in vivo, more complex strategies will be necessary. An example that illustrates the limitation of current models is the fact that, in humans, most of the patients with cancer related to BRCA1 and BRCA2 genetic causal variants are heterozygous, while in contrast, *Brca1* or *Brca2* heterozygous mice are not prone to tumor development.

Despite its pivotal role in HR, the function of the pivotal HR factor RAD51 (see Figure 1B) has yet to be well characterized in vivo. Intriguingly, inactivation of *RAD51* has not been associated with cancer predisposition, and the mechanisms behind this “RAD51 paradox” are still unsolved. The only RAD51 mouse model currently in existence is a RAD51 knockout, which is embryonic lethal, limiting our understanding of RAD51 function in vivo. Therefore, the impact of RAD51 on development and tumorigenesis remains to be investigated in vivo. Importantly, this might present a problem for research programs aiming at designing RAD51 inhibitors as therapeutic compounds [183]. Therefore, the development and use of alternative mouse models addressing the role of RAD51 function in vivo should constitute exciting challenges for future research.

## Figures and Tables

**Table 1:** Embryonic lethal mouse models of complete or severe loss of function of HR genes. Phenotype: when the mutant allele is in homozygosis. EL: embryonic lethal. N/A: not available. “Enumber”: day of embryonic development death.

Gene	Allele	Phenotype	Reference
*Mre11*	*Mre11^Δ5^*	N/A	[80]
*Rad50*	*Rad50^Δ1–2^*	EL: E7	[81]
*Nbs1/Nbn*	*Nbs1^ex1^*	EL: E3-7	[82]
*Nbs1/Nbn*	*Nbs1^ex6^*	EL: E3-7	[83]
*Nbs1/Nbn*	*Nbs1^Δ6^*	EL: N/A	[84]
*CtIP/Rbbp8*	*CtIP^neo^*	EL: E3-7	[85]
*Dna2*	*Dna2^4–7^*	EL: E7	[86]
*Blm*	*Blm^neo^*	EL: E10-14	[87]
*Brca1*	*Brca1^5–6^*	EL: E6-7	[71]
*Brca1*	*Brca1^ko^*	EL: E6-7	[72]
*Brca1*	*Brca1^ex2^*	EL: E6-7	[73]
*Brca1*	*Brca1^223–763^*	EL: E8-13	[74]
*Brca1*	*Brca1* ^−*11*^	EL: E8	[75]
*Brca1*	*Brca1^in10ex11^*	EL: E9	[88]
*Brca1*	*Brca^1700T^*	EL: E10	[89]
*Brca1*	*Brca1^Δ11^*	EL: E12-18	[88]
*Brca1*	*Brca1^Δ2^*	N/A	[90]
*Brca1*	*Brca1^185stop^*	EL: E10-13	[91]
*Brca1*	*BRCA1^5382stop^*	EL: E9-12	[91]
*Brca1*	*Brca1^C61G^*	EL: E12	[92]
*Brca1*	*Brca1^ΔC^*	EL: E9	[93]
*Brca2*	*Brca2* ^−*10*–*11*^	EL: E8-10	[76,94]
*Brca2*	*Brca2^Brdm1^*	EL: E7-8	[78]
*Brca2*	*Brca2^ex11^*	EL: E8	[73]
*Brca2*	*Brca2^1593N-ter^*	EL: E8-9	[95]
*Brca2*	*Brca2^lex2^*	N/A	[96]
*Brca2*	*Brca2^Δ11^*	EL: E8	[97]
*Palb2*	*Palb2^GT^*	EL: E8-9	[98,99]
*Palb2*	*Palb2^Δ2–3^*	EL: E8-10	[100]
*Rad51*	*Rad51^ex5^*	EL: Prior to E8	[69]
*Rad51*	*Rad51^M1^*	EL: E7-8	[70]
*Xrcc2*	*Xrcc2^ex3^*	EL: E12-18	[101]
*Xrcc2*	*Xrcc2^Δ3^*	N/A	[102]
*Rad51b/Rad51l1*	*Rad51b^β-geo^*	EL: E8	[103]
*Rad51c*	*Rad51c^-splice^*	EL: E8-10	[104]
*Rad51c*	*Rad51^Δ2–3^*	EL: E8	[105]
*Rad51d/Rad51l3*	*Rad51d^ex4^* ^–*6*^	EL: E8-11	[106]
*Xrcc3*	*N/A*		
*Pold1*	*Pold1^ko^*	EL: E4-E7	[107]
*Top3a*	*Top3a^ex6^*	EL: prior to E7	[108]

**Table 2:** Nonembryonic lethal mouse models of complete or severe loss of function of HR genes. Phenotype: when the mutant allele is in homozygosis. N/A: not available.

Gene	Allele	Phenotype	Reference
*Exo1*	*Exo1^hyg^*	Lifespan reduction and increased cancer susceptibility	[113]
*Rad54*	*Rad54^307neo^*	No somatic phenotype	[114]
*Sws1*	*Sws1^∆1(A)^*	No somatic phenotype	[115]
*Swsap1*	*Swsap1^∆131^*	No somatic phenotype	[115]
*Mcm8*	*Mcm8^Δ11–12^*	Late onset myeloid tumors	[116,117]
*Mcm9*	*Mcm9^Δ1–2^*	Late onset myeloid tumors	[116,117]
*Hrob*	*Hrob^fs^*	N/A	[54]
*HelQ*	*HelQ^Δc^*	Increased cancer susceptibility	[118]
*Mus81*	*Mux81^ex3^* ^–*5*^	Lifespan reduction and increased cancer susceptibility (lymphoma)	[119]
*Mus81*	*Mus81^Δ9–12^*	No somatic phenotype	[120]
*Eme1*	*Gen1^PB^*	N/A	[121]
*Slx1*	*Slx1^Δ1^*	No somatic phenotype	[122]
*Gen1*	*Gen1^PB^*	Frequently developed kidney and urinary tract defects	[123]
*Slx4/Btbd12*	*Slx4^ko^*	Mice are born in sub-Mendelian ratios and show reduced lifespan and increased cancer susceptibility	[124,125]

**Table 3:** Mouse models of partial loss of function of HR genes. Phenotype in homozygotes.

Gene	Allele	Phenotype	Reference
*Brca1*	*Brca1^tr^*	Develop a variety of tumors: lymphomas, sarcomas and carcinomas/adenomas, including breast carcinomas/adenomas.	[127]
*Brca1*	*Brca1^fl^*	Adult mice present hyperplasia in the gynecologic system, thymic lymphoma and mammary gland hyperplasia with very dense branches and hyperplasic and/or neoplastic foci.	[136]
*Brca1*	*Brca1^SF^*	Increased tumor incidence	[137]
*Brca1*	*Brca1^FH-I26A^*	Normal development, no increased tumor incidence	[137,138]
*Brca1*	*Brca1^S971A^*	Females develop uterus hyperplasia and ovarian abnormalities by 2 years of age.	[139]
*Brca1*	*Brca1^S1152A^*	Some mice exhibit aging-like phenotypes, including growth retardation, skin abnormalities, and delay of the mammary gland morphogenesis. Aging-unaffected 18-month-old females show mammary gland abnormalities.	[140]
*Brca1*	*Brca1^cc^*	Approximately 15% of the expected number of embryos reach birth. The mice who survive until adulthood show developmental features of Fanconi anemia and T cell acute lymphoblastic leukemia.	[141]
*Brca1*	*Brca1^lp^*	Mice are born in the expected Mendelian frequency. These mice show developmental features of Fanconi anemia and T-lymphoblastic lymphoma/leukemia.	[142]
*Brca2*	*Brca2^tm1Cam^*	Mice are born in sub-Mendelian ratios. The extent of embryonic lethality is dependent on the genetic background. The mice who survive until adulthood presented multiple developmental and growth defects, in addition to the development of thymic lymphomas between 12 and 14 weeks of age.	[134]
*Brca2*	*Brca2^Tr2014^*	Mice are born in sub-Mendelian ratios. The extent of embryonic lethality is dependent on the genetic background. The mice who survive until adulthood presented multiple developmental and growth defects, in addition to the development of thymic lymphomas.	[135]
*Brca2*	*Brca2^G25R^*	Increased B-cell lymphoma and other cancer types in aged mice.	[143]
*Brca2*	*Brca2^Δ27^*	Mice are born in sub-Mendelian ratios. The mice that survive until adulthood show an increased incidence of a variety of tumors, including carcinomas, adenomas, lymphomas, and sarcomas.	[144]
*Palb2*	*Palb2^cc6^*	No somatic phenotype.	[145]

**Table 4:** Mouse models of genetic inactivation of HR genes with other DNA repair and DNA damage response genes. Phenotype: when the mutant allele is in homozygosis.

Gene	HR-Mutated Allele	Additional Genetic Modification	Phenotype	Reference
*Brca1*	*Brca1^Δ11^*	*Trp53^+/−^*	Rescue the embryonic lethality. Most female mice present mammary tumors, these mice also present lymphomas and ovarian tumors.	[88]
*Brca1*	*Brca1^Δ11^*	*Trp53^−/−^*	Rescue the embryonic lethality. No further characterization.	[88]
*Brca1*	*Brca1^Δ11^*	*Cdkn1a^−/−^*	No rescue of embryonic lethality	[88]
*Brca1*	*Brca1^Δ11^*	*Bax^−/−^*	No rescue of embryonic lethality	[88]
*Brca1*	*Brca1^Δ11^*	*Atm^−/−^*	Rescue the embryonic lethality. No further characterization.	[149]
*Brca1*	*Brca1^Δ11^*	*Chk2^−/−^*	Rescue the embryonic lethality. These mice present features of premature aging, including decreased skin thickness, kyphosis, decreased bone density and intestinal villi atrophy. In addition, mice exhibit enhanced cancer prevalence, including mammary and ovary tumors in high frequency.	[149]
*Brca1*	*Brca1^lp^*	*Trp53^−/−^*	Rescue of bone marrow failure, but not growth defects. These mice show increased frequency of T-lymphoblastic lymphoma/leukemia	[142]
*Brca1*	*Brca1^Δ11^*	*Trp53bp1^−/−^*	Rescue of embryonic lethality. No increased tumor incidence or features of premature aging.	[147]
*Brca1*	*Brca1^Δ11^*	*H2ax^−/−^*	No rescue of embryonic lethality.	[150]
*Brca1*	*Brca1^Δ11^*	*Rnf168^−/−^*	No rescue of embryonic lethality.	[150]
*Brca1*	*Brca1^Δ11^*	*Rnf8^−/−^*	No rescue of embryonic lethality.	[150]
*Brca1*	*Brca1^Δ11^*	*Trp53bp1^S25A^*	Rescue of embryonic lethality. Features of premature aging and decreased lifespan.	[151]
*Brca1*	*Brca1^Δ11^*	*Ku80^−/−^*	No rescue of embryonic lethality	[152]
*Brca1*	*Brca1^ex2^*	*Trp53bp1^−/−^*	Pups were obtained at a frequency slightly lower than the expected Mendelian ratio.	[152]
*Brca1*	*Brca1^ΔC^*	*Trp53bp1^−/−^*	Rescue of embryonic lethality. These mice develop thymic lymphomas between 3-6 months of age.	[141]
*Brca1*	*Brca1^Δ5–13^*	*Trp53bp1^−/−^*	Partial rescue of embryonic lethality. These mice develop thymic lymphoma within 7 months	[148]
*Brca2*	*Brca2^Δ27^*	*Trp53^+/−^*	Decrease in lifespan and increase in tumor prevalence.	[153]
*Brca2*	*Brca2^G25R^*	*Trp53^+/−^*	Enhanced tumor prevalence.	[143]
*Palb2*	*Palb2^cc6^*	*Rnf168^−/−^*	Embryonic lethal.	[150]
